# Landslide Sensitivity and Response to Precipitation Changes in Wet and Dry Climates

**DOI:** 10.1029/2022GL099499

**Published:** 2022-07-06

**Authors:** Alexander L. Handwerger, Eric J. Fielding, Simran S. Sangha, David P. S. Bekaert

**Affiliations:** ^1^ Jet Propulsion Laboratory California Institute of Technology Pasadena CA USA; ^2^ Joint Institute for Regional Earth System Science and Engineering University of California, Los Angeles Los Angeles CA USA; ^3^ Earth, Planetary, and Space Sciences University of California, Los Angeles Los Angeles CA USA

**Keywords:** landslides, InSAR, remote sensing, open‐access data, climate, California

## Abstract

Slow‐moving landslides are hydrologically driven. Yet, landslide sensitivity to precipitation, and in particular, precipitation extremes, is difficult to constrain because landslides occur under diverse hydroclimatological conditions. Here we use standardized open‐access satellite radar interferometry data to quantify the sensitivity of 38 landslides to both a record drought and extreme rainfall that occurred in California between 2015 and 2020. These landslides are hosted in similar rock types, but span more than ∼2 m/yr in mean annual rainfall. Despite the large differences in hydroclimate, we found these landslides exhibited surprisingly similar behaviors and hydrologic sensitivity, which was characterized by faster (slower) than average velocities during wetter (drier) than average years, once the impact of the drought diminished. Our findings may be representative of future landslide behaviors in California where precipitation extremes are predicted to become more frequent with climate change.

## Key Points

- Open‐access standardized products can be used to identify and monitor landslides over large regions
- Slow‐moving landslides occur in both dry and wet environments with mean annual rainfall ranging from ∼200 to ∼2000 mm/yr
- Landslides are sensitive to seasonal, annual, and multi‐year changes in rainfall in both dry and wet environments

## 1 Introduction

A foundational paradigm in landslide science is that precipitation triggers landslides. Precipitation promotes slope instability as water flows through the ground and raises the water table, or creates perched water tables, and as a result, increases pore‐water pressures, reduces the effective normal stress (normal stress minus pore‐water pressure), and reduces the frictional strength of the hillslope (Bogaard & Greco, 2016; Terzaghi, 1951). Once a hillslope fails as a landslide it can accelerate rapidly and fail catastrophically (Iverson et al., 2015; Jibson, 2006; Shugar et al., 2021), move downslope slowly for years to hundreds of years (Mackey et al., 2009; Nereson & Finnegan, 2019; Rutter & Green, 2011), or move slowly for a period of time before stabilizing or failing catastrophically (Agliardi et al., 2020; Handwerger, Huang, et al., 2019; Iverson, 2005; Kilburn & Petley, 2003). These different behavioral modes have important consequences for hazard assessment because fast‐moving landslides can move at rates up to tens of meters per second and can easily claim lives (Iverson et al., 2015; Shugar et al., 2021), while slow‐moving landslides move at rates of meters per year or less and can damage infrastructure (Lacroix, Handwerger, et al., 2020; Merriam, 1960).

Persistently active slow‐moving landslides are well‐suited for exploring hydrologic controls on landslide motion because they are relatively easy to monitor (compared to landslides with catastrophic failures), occur in wet and dry environments around the world where water is delivered by rainfall (Bayer et al., 2018; Malet et al., 2002), snowmelt (Coe et al., 2003; Matsuura et al., 2008), or irrigation (Lacroix, Dehecq, et al., 2020; Merriam, 1960), and their motion is closely linked to local groundwater conditions (Corominas et al., 2005; Iverson & Major, 1987; Murphy et al., 2022). Furthermore, the hydrologic controls on slow‐moving landslides, via pore pressure changes, are akin to the hydrologic controls on faults (Bhattacharya & Viesca, 2019; Cappa et al., 2019), glaciers (Minchew & Meyer, 2020; Moon et al., 2014), and rock glaciers (Cicoira et al., 2019; Kenner et al., 2017), and therefore investigating these landslides allows us to better understand each system.

Previous investigations on the hydrologic controls on slow‐moving landslides have shown that precipitation causes slow‐moving landslides to accelerate once the pore‐water pressures have increased to sufficient levels in the landslide body and decelerate when the pore‐water pressures drop (Finnegan et al., 2021; Iverson & Major, 1987; Malet et al., 2002). Thus, slow‐moving landslides can slow down or stop moving during dry periods, and speed up, reactivate, or fail catastrophically during wet periods (Bennett, Roering, et al., 2016; Handwerger, Fielding, et al., 2019; McSaveney & Griffiths, 1987; Nereson & Finnegan, 2019). The hydrologic response of landslides can also be size‐dependent where larger and thicker landslides are somewhat less sensitive to daily to annual changes in rainfall compared to smaller and thinner landslides that typically experience greater swings in pore‐water pressure (Bennett, Roering, et al., 2016; Handwerger, Fielding, et al., 2019). Indeed, this is an expected consequence of pore water transmission in saturated ground (Iverson & Major, 1987). However, recent work by Finnegan et al. (2021) shows nearly instantaneous pore pressure transmission to depth once the vadose zone of the Oak Ridge landslide, California becomes saturated each year, which suggests vadose zone thickness, rather than total landslide thickness, may be the relevant length scale controlling the landslide response in settings where the surface of a landslide becomes unsaturated, for example, in locations with highly seasonal rainfall delivery. Nonetheless, both climate and landslide size may govern the hydrologic sensitivity of landslides.

Satellite‐based interferometric synthetic aperture radar (InSAR) data can be analyzed alongside precipitation and groundwater data and used to inventory and monitor landslides with the high spatial and temporal resolution necessary to explore hydrologic controls on landslide motion (Bayer et al., 2018; Cohen‐Waeber et al., 2018; Handwerger et al., 2013). The open‐access data collected by Copernicus Sentinel‐1 A/B satellites, in particular, has revolutionized InSAR studies on landslides (Bayer et al., 2018; Carlà et al., 2019; Handwerger, Huang, et al., 2019; Intrieri et al., 2017; Liu et al., 2021; Raspini et al., 2018), and other ground surface deformation (Cigna & Tapete, 2021; Huang et al., 2017; Lundgren et al., 2020; Strozzi et al., 2020), and has led to the development of automated InSAR processing systems that produce derived higher‐level standard products that can be used for scientific research (Buzzanga et al., 2020; Dehls et al., 2019; Jones et al., 2021; Lazecký et al., 2020). These derived standard products will become especially important as the volume of InSAR data continues to grow, making it increasingly challenging to process and download InSAR data for large regions on a personal computer. Furthermore, the recent push to provide open‐access standardized InSAR products, along with a suite of tools to analyze these data (e.g., Morishita et al., 2020; Yunjun et al., 2019), increases data accessibility to the broader geoscience community, which will undoubtedly lead to major scientific advances.

**Figure 1:**
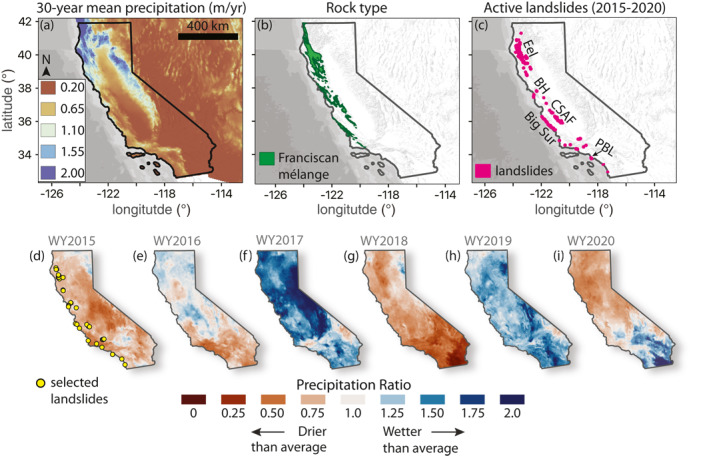
Maps of precipitation, rock type, and landslide locations. (a) 30‐year mean water year precipitation (m/yr) with period WY1990‐WY2019 calculated from Parameter‐elevation Regressions on Independent Slopes Model data. (b) Simplified geologic map showing the areal extent of the Franciscan mélange rock unit. (c) Location of active landslides identified with our interferometric synthetic aperture radar analyses. Well‐studied landslide groups labeled Eel = Eel River, BH = Berkeley Hills, CSAF = Central San Andreas Fault, PBL = Portuguese Bend landslide. (d–i) Precipitation Ratio (total WY precipitation/30‐year mean precipitation) for WY2015‐WY2020. Red colors correspond to drier than average years and blue colors correspond to wetter than average years. Yellow circles in (d) show landslides selected for detailed time series analyses.

In this study we analyze open‐access standardized Sentinel‐1 interferograms automatically processed by the JPL‐Caltech Advanced Rapid Imaging and Analysis (ARIA) Center for Natural Hazards project (Bekaert et al., 2019) to identify and monitor landslides in both wet and dry climates in California, USA. California has a large quantity of active slow‐moving landslides and has been a major focus area for landslide investigations for decades (Iverson & Major, 1987; Keefer & Johnson, 1983; Kelsey, 1978; Merriam, 1960). Slow‐moving landslides in California exhibit distinct seasonal kinematic patterns (Cohen‐Waeber et al., 2018; Finnegan et al., 2021; Handwerger et al., 2013; Iverson & Major, 1987) that are a consequence of the regions Mediterranean climate with mild wet winters and hot dry summers, and multi‐year kinematic changes that result from precipitation deficits or surplus (Bennett, Roering, et al., 2016; Booth et al., 2020; Mackey et al., 2009; Nereson & Finnegan, 2019). California also has a large rainfall gradient from north to south and west to east, with parts of northern California receiving >3,000 mm/yr of rainfall and parts of southern California receiving <200 mm/yr (Figure 1a). There are slow‐moving landslides in both wet and dry regions of California, which presents an opportunity to examine how variability in hydroclimatology controls landslide behaviors.

We focus our study between the 2015 and 2020 water years (WY), during which California experienced extreme changes in rainfall (Figures 1d–1i and Figure S1 in Supporting Information S1). WY2015 and WY2016 were the last two years of a historic drought, which was one of the most severe for hundreds of years (Robeson, 2015; Swain et al., 2014). The drought officially ended in WY2017, which was an extremely wet year across most of California, and was the second wettest year on record in places (Swain et al., 2018; Wang et al., 2017). There were many landslides that were triggered or accelerated and reactivated in WY2017 (Finnegan et al., 2021; Handwerger, Fielding, et al., 2019), including the catastrophic Mud Creek landslide that destroyed State Highway 1 (Handwerger, Huang, et al., 2019; Jacquemart & Tiampo, 2021; Warrick et al., 2019). Dry conditions returned in WY2018 due to below average rainfall, followed by wet conditions in WY2019 due to above average rainfall, and finally a return to dry conditions in WY2020 due to below average rainfall. These back and forth changes from dry to wet conditions are consistent with long‐term climate predictions and therefore may be representative of climate patterns in California over the next century (Persad et al., 2020; Polade et al., 2017; Swain et al., 2018).

## 2 Data and Methods

### 2.1 Interferometric Synthetic Aperture Radar Processing and Analysis

Open‐access SAR data from the C‐band (∼5.6 cm radar wavelength) Copernicus Sentinel‐1 A/B satellites were automatically processed to standardized geocoded, unwrapped interferograms (GUNW) by the ARIA project (Bekaert et al., 2019). ARIA uses the open‐source JPL InSAR Scientific Computing Environment (ISCE) software to process the interferograms (Rosen et al., 2012). These standardized interferograms are corrected for topographic contributions to phase and geocoded to a ∼90 m (3 arc s) pixel spacing using the Shuttle Radar Topography Mission (SRTM) digital elevation model (DEM) (Farr et al., 2007). ARIA provides key data needed for deformation analyses and time series inversions including geocoded unwrapped interferograms, coherence, incidence and azimuth angles, and the SRTM DEM and water mask.

We used the ARIA‐tools open‐source package in Python (Buzzanga et al., 2020) to download and prepare 1689 interferograms covering California (Table S1). We inverted the interferograms to deformation time series using the Miami InSAR Time‐series software in PYthon (MintPy) (Yunjun et al., 2019). To remove longer time span interferograms that often have low coherence and are more likely to contain unwrapping errors for persistently moving features such as slow‐moving landslides (e.g., Handwerger, Huang, et al., 2019), we used only 2 consecutive interferogram pairs in the time series inversion. We quantified InSAR uncertainty using a bootstrapping technique (Bekaert et al., 2020; Efron & Tibshirani, 1986) with 400 iterations for each time series. More information on the InSAR data processing can be found in the Supporting Information S1.

### 2.2 Landslide Reconnaissance and Metrics

We identified active landslides by examining the 5‐year time‐averaged InSAR velocity maps. Active slow‐moving landslides displayed localized deformation zones with relatively high velocity (Figures S2 and S3 in Supporting Information S1). We then confirmed that the InSAR signals corresponded to true landslides by overlaying the InSAR velocity maps onto DEMs, Google Earth imagery, and previously published landslide inventories. We began searching for active landslides by examining the InSAR velocity in well‐known landslide areas in northern California (Bennett, Miller, et al., 2016; Handwerger, Fielding, et al., 2019; Kelsey, 1978), central California (Booth et al., 2020; Cohen‐Waeber et al., 2018; Finnegan et al., 2019; Scheingross et al., 2013; Wills et al., 2001), and southern California (Calabro et al., 2010; Jibson, 2006; Merriam, 1960; Swirad & Young, 2021; Young, 2015). We also examined the InSAR data alongside the California Geologic Survey statewide landslide inventory (Wills et al., 2017). After examining these known landslide areas, we then systematically expanded outward from these regions to identify active landslides in all mountainous regions of California. We quantified landslide metrics such as area, length, width, and slope angle using the SRTM ∼30 m (1 arc s) DEM.

To estimate landslide thickness (and volume), which is often thought to be one of the key length scales that controls landslide response to rainfall (e.g., Handwerger et al., 2013), we applied recently developed geometric scaling relations for slow‐moving landslides in California (Handwerger et al., 2021). These are particularly useful for estimating thickness of slow‐moving landslides, which is difficult to do without numerous ground‐based instruments. Landslide scaling relations take the form of a power function as

(1) $$h\;=\;c_{h}A^{\zeta }\text{and}\;V\;=\;c_{V}A^{\gamma }$$

where *A* is the landslide area, *h* is the estimated mean thickness, *V* is the estimated volume, γ and *ζ* are the scaling exponents and $c_{V}$ and $c_{h}$ are fit intercepts (see parameters in Table S2 in Supporting Information S1). Landslide geometric scaling relations also vary by landslide type and material (Bunn et al., 2020; Handwerger et al., 2021; Larsen et al., 2010). To apply the most appropriate scaling relations (Handwerger et al., 2021), we classified slow‐moving landslides as slumps (one primary kinematic zone and low length/width aspect ratios), earthflows (one primary kinematic zone and medium aspect ratios), and landslide complexes (amalgamations of landslides with multiple kinematic zones and high aspect ratios).

To further assess the kinematic behavior of the slow‐moving landslides in wet and dry environments during wet and dry years, we selected a subset of landslides to perform detailed time series investigation (Figure 1d). These landslides were selected based on their relatively high velocity signal (i.e., strong InSAR signal) and their location within California's different hydroclimatic regimes. We characterized the landslide motion by calculating the spatial mean of the fastest moving kinematic zone and used a moving median temporal smoothing filter to further reduce noise and highlight the seasonal and annual deformation signals (Figures S2 and S3 in Supporting Information S1). We explored environmental controls on landslides by examining the rock type and precipitation data in active landslide areas. Rock type data are provided by the California Geologic Survey (Jennings et al., 2010) and precipitation data are provided by the Parameter‐elevation Regressions on Independent Slopes Model (PRISM) (see Open Research). We then quantified landslide sensitivity to rainfall by exploring relative changes in precipitation and landslide velocity. To explore relative changes in precipitation and velocity, we defined the Precipitation Ratio as the total water year precipitation divided by the 30‐year mean water year precipitation (calculated from WY1990‐WY2019) at each landslide (Figures 1d–1i), and the Velocity Ratio as the water year velocity divided by the average velocity from WY2016‐WY2019.

## 3 Results

### 3.1 Landslide Inventory

We manually identified and mapped 247 active slow‐moving landslides in California (Figure 1c). Many, if not all of these landslides have been previously identified by other studies (Bennett, Miller, et al., 2016; Cohen‐Waeber et al., 2018; Finnegan et al., 2019; Handwerger, Fielding, et al., 2019; Jibson, 2006; Kelsey, 1978; Merriam, 1960; Scheingross et al., 2013; Swirad & Young, 2021; Wills et al., 2001). These landslides consisted of different types including 71 slumps, 72 earthflows, and 104 landslide complexes (Table S3). As an expected consequence of the relatively coarse resolution (90 m pixel spacing) of the ARIA standardized InSAR product, we identified mostly larger landslides with areas ranging from 0.018 to 11 km^2^ with a mean area of 0.5 km^2^ (Table S3). The active landslides are distributed throughout the mountainous regions in western California, with the vast majority (230 of 247) in the Coast Ranges, and they spanned nearly the entire latitudinal extent of the state. Regions with the highest density of landslides include the well‐known landslide hotspots such as the Eel River catchment, Big Sur coast, and Central San Andreas Fault (Figure 1c). There are also landslides located in populated and highly traveled zones such as Los Angeles and Berkeley, and along California State Highway 1 and State Highway 101 and thus pose a threat to infrastructure and life. Although we identified a large quantity of landslides, our inventory is an underestimate of the true landslide activity in California for three main reasons; (a) we were unable to identify landslides in regions with high seasonal snow cover (e.g., Sierra Nevada Mountains). To better explore regions with seasonal snow requires a different InSAR processing strategy that only utilizes data from snow‐free periods. (b) The coarse resolution prevents us from imaging many of the smaller landslides that have been identified with higher resolution InSAR or field data (e.g., Handwerger, Fielding, et al., 2019; Kelsey, 1978; Nereson & Finnegan, 2019; Scheingross et al., 2013; Schulz et al., 2018). And (c) InSAR provides a 1D measurement and cannot detect landslide motion in the direction of the satellite heading (i.e., observational bias).

Despite the large variability in rock type throughout California (Figure S4 in Supporting Information S1), we found that 228 of 247 landslides occurred in host rocks broadly defined as marine and nonmarine sedimentary or metasedimentary rock units (Figure S4 in Supporting Information S1 and Table S3). Of these landslides, 176 (71% of total) landslides are hosted within the Franciscan complex mélange (Figure 1b), which indicates a strong lithologic control on the distribution of slow‐moving landslides. Numerous recent studies have made similar findings indicating that rock type exerts a primary control on slow‐moving landslides in California (Bennett, Miller, et al., 2016; Handwerger, Fielding, et al., 2019; Scheingross et al., 2013; Xu et al., 2021) and throughout the world (see refs. in Lacroix, Handwerger, et al., 2020).

Estimates of precipitation from PRISM data showed that active landslides are occurring in both wet and dry environments (Figure 1). We found more than an order of magnitude precipitation variation between the wettest active landslide in northern California (30‐year mean = 2180 mm/yr) and driest landslide in southern California (30‐year mean = 216 mm/yr) (Figure 1a).

### 3.2 Seasonal and Annual Landslide Behavior

**Figure 2:**
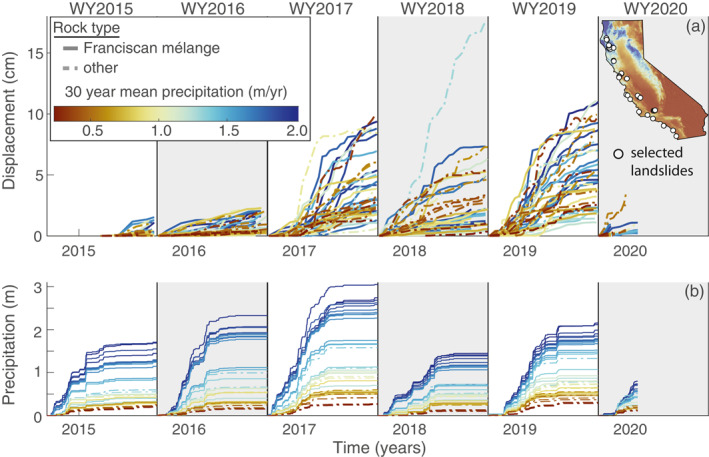
Landslide and precipitation time series for 38 selected landslides in California. (a) Cumulative displacement time series projected onto the downslope direction and separated by water year (WY). The time series for each landslide are smoothed using a moving median temporal filter. Solid lines correspond to landslides occurring within the Franciscan mélange rock unit. Colors correspond to 30‐year mean water year precipitation (WY1990‐WY2019) for each landslide. Inset shows the location of the selected landslides on the 30‐year mean precipitation map. (b) Cumulative precipitation time series for each landslide separated by WY and colored by 30‐year mean WY precipitation.

To assess the behavior of landslides occurring in wet and dry climates, we selected a subset of 38 landslides spanning more than an order of magnitude in 30‐year mean precipitation (Figure 2 and Table S4). The subset of landslides consisted of different landslide types, rock types, and occurred in different environments including coastal and inland regions, as well as developed and undeveloped areas.

Annual downslope velocities ( ±  bootstrap uncertainty) averaged over the full study period ranged from 0.85 ± 0.56 to 9.7 ± 1.2 cm/yr (Table S4). Landslides occurring in both wet and dry regions of California exhibited seasonal kinematic changes in response to seasonal precipitation each year (Figure 2). Each landslide accelerated in response to infiltrating rainfall during the wet season before decelerating back to lower rates, or completely stopping, during the dry season.

**Figure 3:**
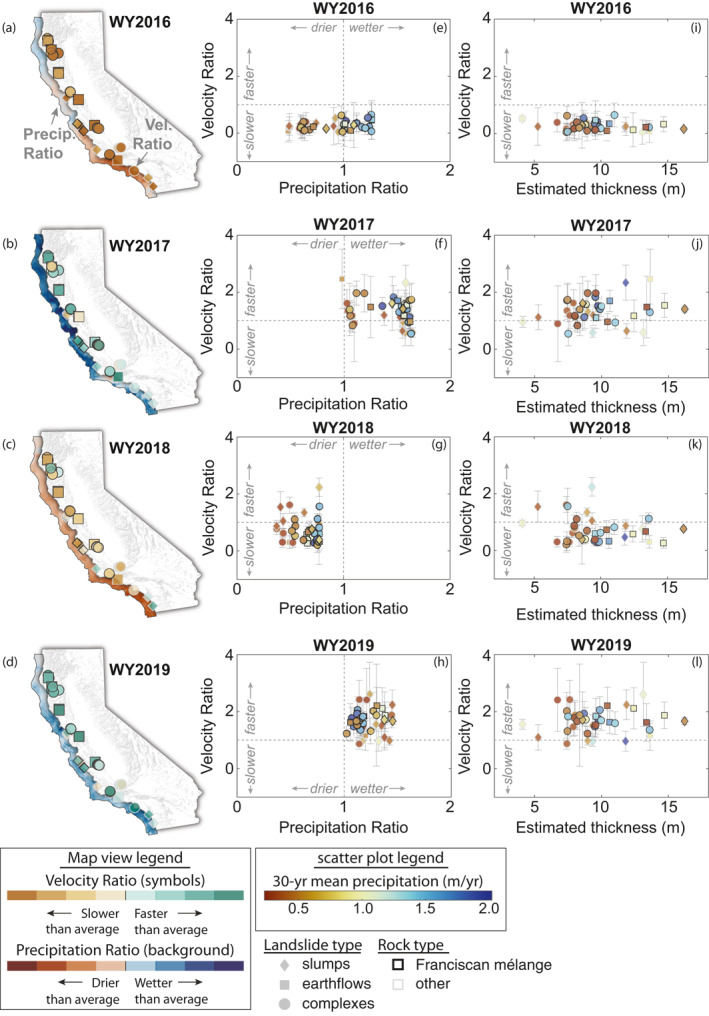
Landslide response to changes in precipitation. (a–d) Maps of velocity and precipitation ratio by water year. Brown to green color symbols correspond to velocity ratio values for each landslide. Red to blue colors in background correspond to precipitation ratio. Symbols correspond to landslide type. Rock type is shown by black or gray symbol border color. (e–h) Velocity ratio as a function of precipitation ratio for selected landslides. (i–l) Velocity ratio as a function of estimated landslide thickness. Error bars show the uncertainty in the velocity ratio. Red to blue colors correspond to the 30‐year mean water year precipitation (WY1990‐WY2019) for each landslide. Symbols correspond to landslide type. Rock type is shown by black or gray symbol border color. We calculated the velocity ratio uncertainty using standard error propagation and assumed nil uncertainty in the precipitation data.

The landslides also responded to changes in seasonal rainfall each year. We found large changes in seasonal precipitation caused large changes in displacement (Figures 2 and 3). While there is no clear relationship between velocity, precipitation, and landslide size (Figure 3 and S5 in Supporting Information S1), the landslides moved faster than average during the wetter WY2017 and WY2019, and slower than average during the drier WY2016 and WY2018 (Figure 3). Interestingly, we observed the largest displacement at a single landslide during the drier than average WY2018 (Figure 2). This landslide is located on the coast and is likely subject to other driving forces such as debuttressing from wave erosion at its toe (see location of “landslide 22” in Table S4). However, we cannot constrain additional driving forces with our data set. While we did detect several other coastal landslides, most of their motion appears to be driven by rainfall (Figure 2).

Landslide sensitivity to precipitation also appeared to change during the study period. For instance, WY2016, which was the final year of the historic California drought, had both wetter and drier than average conditions in certain places, however all of the landslides were moving slower than average (velocity ratio <1). WY2018 was drier than WY2016 across California, but a few landslides exhibited velocity ratios >1. Our findings suggest that antecedent rainfall from the previous wet seasons, particularly the lingering impact of long‐term droughts, likely play an important control on landslide behavior and sensitivity to rainfall.

## 4 Discussion and Conclusion

Our study documents the first application of open‐access standardized InSAR products from JPL ARIA to identify and monitor landslides across large regions. Although the coarse resolution of the ARIA InSAR product limits our ability to detect smaller landslides, it still provides valuable data that can be used to better understand landslide processes. Due to the large volume of open‐access InSAR data that is currently available, and will continue to increase with time, especially with the upcoming launch of the NASA‐ISRO SAR (NISAR) satellite, standardized InSAR products will become one of the primary ways to deliver InSAR data to the broader scientific community. The JPL Observational Products for End‐Users from Remote Sensing Analysis (OPERA) project will soon be generating an operational high resolution displacement timeseries from Sentinel‐1 and NISAR data over North America. With a spatial resolution of 30 m or better this product will be well‐suited for identifying and monitoring landslides. Thus, it is important to continue to explore new approaches to analyze these InSAR products for scientific research, including use of automated or semi‐automated detection and mapping techniques (e.g., Amatya et al., 2021; Milillo et al., 2021).

Active slow‐moving landslides across California occur in both wet and dry environments. Despite more than an order of magnitude difference in mean annual rainfall and factor of ∼4 difference in estimated landslide thickness, these landslides exhibit similar first order behaviors including (a) seasonal and annual changes in displacement corresponding to local changes in rainfall, and (b) sensitivity to seasonal, annual, and multi‐annual changes in rainfall. One explanation for climate‐ and size‐independent sensitivity is that persistently active landslides maintain sufficiently high groundwater levels that keep them close to an acceleration threshold. Prior work has shown that active slow‐moving landslides typically have high groundwater levels year round and become effectively saturated during the wet season (Finnegan et al., 2021; Iverson & Major, 1987; Malet et al., 2002; Schulz et al., 2009, 2018). The ability to maintain high groundwater levels may be a consequence of rock type‐controlled critical zone (i.e., ground surface to unweathered bedrock) structure (Murphy et al., 2022). Hahm et al. (2019) showed that landslides in a wet region of northern California (mean annual rainfall ∼1800 mm/yr) in the Franciscan mélange, the predominant bedrock of landslides in our inventory, have a critical zone characterized by a thin (<3 m) seasonally unsaturated zone with low hydraulic conductivity that becomes effectively saturated after ∼100–200 mm of seasonal rainfall. Similarly, Finnegan et al. (2021), showed that the Oak Ridge landslide, in an area of moderate rainfall in central California (mean annual rainfall ∼640 mm/yr), also becomes effectively saturated after ∼200 mm of seasonal rainfall. While not all landslides in our study occur in the Franciscan mélange, the other rock types are mostly marine and nonmarine sedimentary rocks that may bear similarity and thus may have a similar critical zone structure (Riebe et al., 2017). Additionally, the landslides themselves may create environments that allow water retention due to low permeability shear zones that inhibit water flow (Baum & Reid, 2000; Nereson et al., 2018). Therefore, landslides with thin seasonally unsaturated zones can often reach saturation in both the wetter and drier parts of the state. Once saturation occurs, excess precipitation should be shed as overland flow (e.g., Hahm et al., 2019), which may explain why landslides exhibit a muted response to large differences in annual rainfall across California (e.g., Murphy et al., 2022).

Another consequence of limited annual groundwater fluctuations is that slow‐moving landslides display a relatively narrow range of velocities and rarely fail catastrophically. Finnegan et al. (2021) proposed that the narrow range of pore‐water pressures (10–20 kPa) experienced by slow‐moving landslides provides a natural limit to the stress changes that drive landslide acceleration. Yet, slow‐moving landslides occasionally fail catastrophically. One example is the 2017 Mud Creek landslide in California (also in Franciscan mélange) that exhibited a minimum of 8 years of slow motion prior to rapid acceleration and collapse (Handwerger, Huang, et al., 2019). This transition from stable to unstable sliding may result from additional mechanisms that cause pore‐water pressures to rise beyond the maximum from rainfall inputs. Both shear‐induced contraction and longitudinal compression of landslide material can cause pore‐water pressures to increase sharply (e.g., Iverson, 2005; Iverson et al., 2015), as can weakening mechanisms, such as slip localization (e.g., Handwerger, Huang, et al., 2019; Viesca & Rice, 2012). Although many mechanical‐hydrological interactions contribute to the behavior of landslides, our results agree with Finnegan et al. (2021) and Murphy et al. (2022), which suggest that the volume of material that can accommodate water input exerts a primary control on the landslide sensitivity and response to precipitation.

Our study revealed that active slow‐moving landslides moved seasonally during both dry and wet years and in dry and wet climates, indicating that even during dry periods and at dry landslides, there is often still sufficient water input to maintain downslope motion for many landslides. Climate models predict that rainfall in California is likely to become more seasonal (i.e., a higher proportion of rainfall delivered in December to March) (Swain, 2021) and dry to wet year extremes will become more common (Dong et al., 2019; Persad et al., 2020; Polade et al., 2017; Swain et al., 2018). Therefore, our study period may be representative of future precipitation and landslide behavioral patterns throughout California. While we currently cannot reliably predict landslide motion due to complex nonlinear relationships between precipitation, pore‐water pressure, and velocity (e.g., Carey et al., 2019; Malet et al., 2002; Murphy et al., 2022), we may be able to predict relative changes in landslide velocity in response to relative changes in precipitation. Therefore, it is necessary to continue to document landslide behaviors during “normal” years that may serve as baselines for comparison and prediction of future landslide behaviors.

## Supporting information

Supporting Information S1

Table S1

Table S2

Table S3

Table S4

## Data Availability

ARIA standard products were downloaded from the ASF Distributed Active Archive Centers (DAAC) using ARIA‐tools available at https://github.com/aria-tools/ARIA-tools. The full list of interferograms used are shown in Table S1. The landslide inventoriy metrics are included in the Supporting Information (Table S3 and S4) and shapefile polygons are archived in Zenodo (https://doi.org/10.5281/zenodo.6780616). Precipitation data is provided by Parameter‐elevation Regressions on Independent Slopes Model (PRISM) available at https://prism.oregonstate.edu/. Precipitation time series for each landslide is taken at the centroid location listed in Table S4. The Miami INsar Time‐series software in PYthon (MintPy) is available at https://github.com/insarlab/MintPy. Shuttle Radar Topography Mission (SRTM) DEMS are available at https://www.usgs.gov/centers/eros. DEM tiles used in this study are listed in Table S6. Lidar digital elevation models used in Figure S2 in Supporting Information S1 inset are provided by OpenTopography (data set name: USGS LPC CA LosAngeles 2016 LAS 2018) and may be downloaded online (http://www.opentopography.org).
