# Supplementary material for: Landslide Sensitivity and Response to Precipitation Changes in Wet and Dry Climates
Source: Geophys Res Lett. 2022 Jul 6;49(13):e2022GL099499. doi: 10.1029/2022GL099499 (PMC9540568; doi:10.1029/2022GL099499)
Supplement: Supplementary file 1 — Supporting Information S1 [file GRL-49-e2022GL099499-s001.pdf]

Geophysical Research Letters

Supporting Information for

Landslide Sensitivity and Response to Precipitation Changes in Wet and Dry Climates

Alexander L. Handwerger[1,2], Eric J. Fielding[1], Simran S. Sangha[1,3], and David P.S. Bekaert[1]

[1]Jet Propulsion Laboratory, California Institute of Technology, Pasadena, CA, USA. [2]Joint Institute for Regional Earth System Science and Engineering, University of California, Los Angeles, Los Angeles, CA, USA. [3]Earth, Planetary, and Space Sciences, University of California, Los Angeles, Los Angeles, CA, USA.

Contents of this file

Text S1
Figures S1 to S5
Tables S2 and S5

Additional Supporting Information (Files uploaded separately)

Captions for Tables S1, S3, S4, and S6

© 2022. California Institute of Technology. Government sponsorship acknowledged.

Text S1.

InSAR Processing and Analyses

JPL-Caltech ARIA automatically processed standardized interferograms with Sentinel-1 data between 2015 and 2021 for California. At the time this study was performed however the InSAR data were only processed up to early 2020 (Figures 2). ARIA produced interferograms with 3 nearest connected neighbors and year-long pairs, however we only used the 2 nearest connected neighbors in our time series inversions. We used the ARIA-tools open-source package in Python (https://github.com/aria-tools) to download all of the interferograms covering California. We downloaded 13825 individual products which was equivalent to 1689 merged interferograms from ascending tracks 35, 64, and 137, and descending tracks 42, 71, 115, 144, 173 (the full list of the InSAR data used in this study are in Table S1). The ARIA-tools package combines adjacent products into merged interferograms. We found there were sometimes discontinuities in the merged interferograms that resulted during unwrapping between data frames. Interferograms containing discontinuities were manually identified and removed from our analyses for the landslide identification stage, but were included for the time series analyses of selected landslides because the individual landslides did not span these discontinuities.

In order to search for landslides, it is important to use local reference points to help further reduce long wavelength noise that can obscure the landslide signal. We selected 32 regional stable (i.e., no motion) reference points that were used to reset the InSAR data velocity values (Table S5) and facilitate landslide detection. Additionally, we removed noise by excluding pixels with coherence less than 0.4. To further improve the landslide signal, we applied linear deramping, DEM error correction (Fattahi & Amelung, 2013), and tropospheric corrections (Jolivet et al., 2011) with the European Centre for Medium-Range Weather Forecasts (ECMWF) ERA-5 reanalysis data set.

Once we selected the 38 landslides for time series analyses, we subset both ascending and descending InSAR data for each landslide and reprocessed the time series using a new local stable reference point (Table S4). We then selected either ascending or descending data, depending on which data showed the best quality landslide signal (Figure S2). Finally, we projected the line-of-sight time series onto the mean downslope direction of each landslide, assuming surface-parallel motion using:

$$D_{downslope} = \frac{D_{LOS}}{sin(\alpha - \beta)sin(\theta_{inc})sin(\theta_{slp}) + cos(\theta_{inc})sin(\theta_{slp})}$$

where α is the heading direction (in degrees, positive counterclockwise from north) of the radar platform in the horizontal plane, θ_{inc} is the incidence angle, β is the mean azimuth angle of the landslide (i.e., downslope direction heading) and θ_{slp} is the mean hillslope angle of the landslide (Liu et al., 2013). This downslope projection can provide more accurate estimates of the true landslide displacement magnitude.

© 2022. California Institute of Technology. Government sponsorship acknowledged.

Supplementary Figures

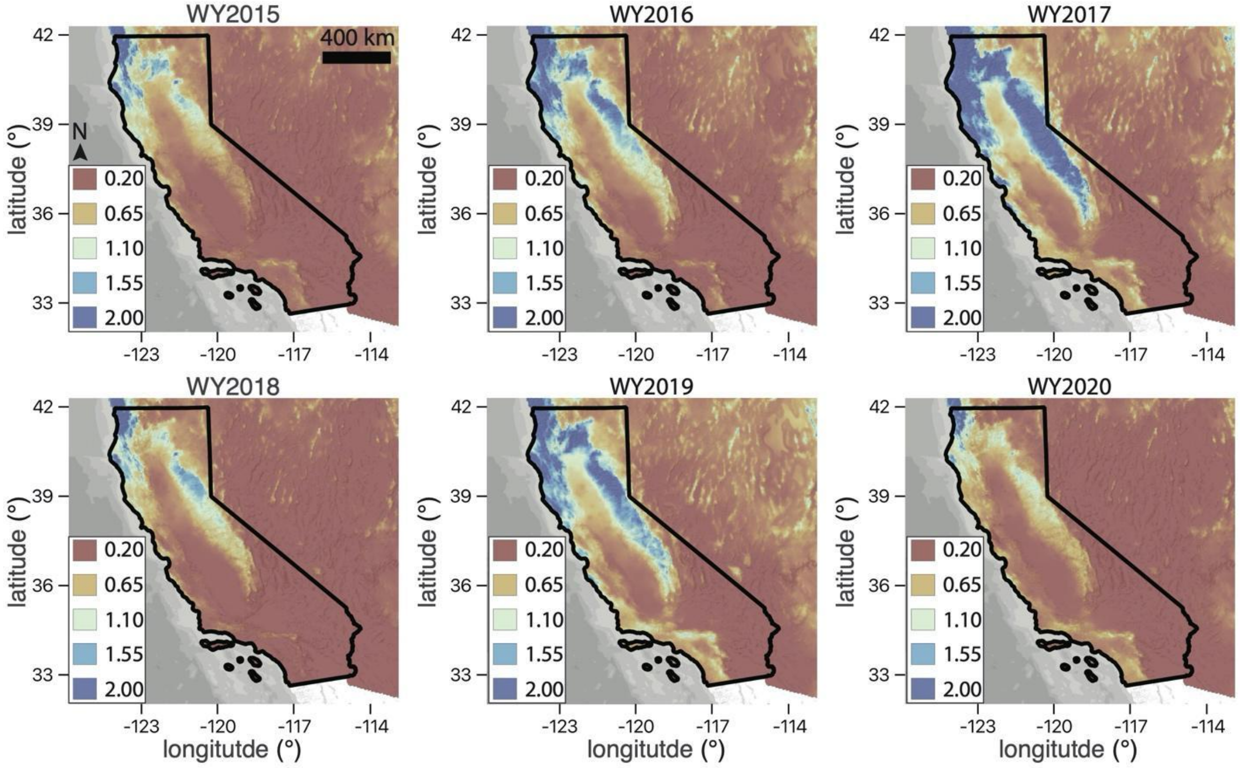

Figure S1. Water year precipitation maps. Colors show the total water year precipitation (m/yr). Data are from PRISM.

© 2022. California Institute of Technology. Government sponsorship acknowledged.

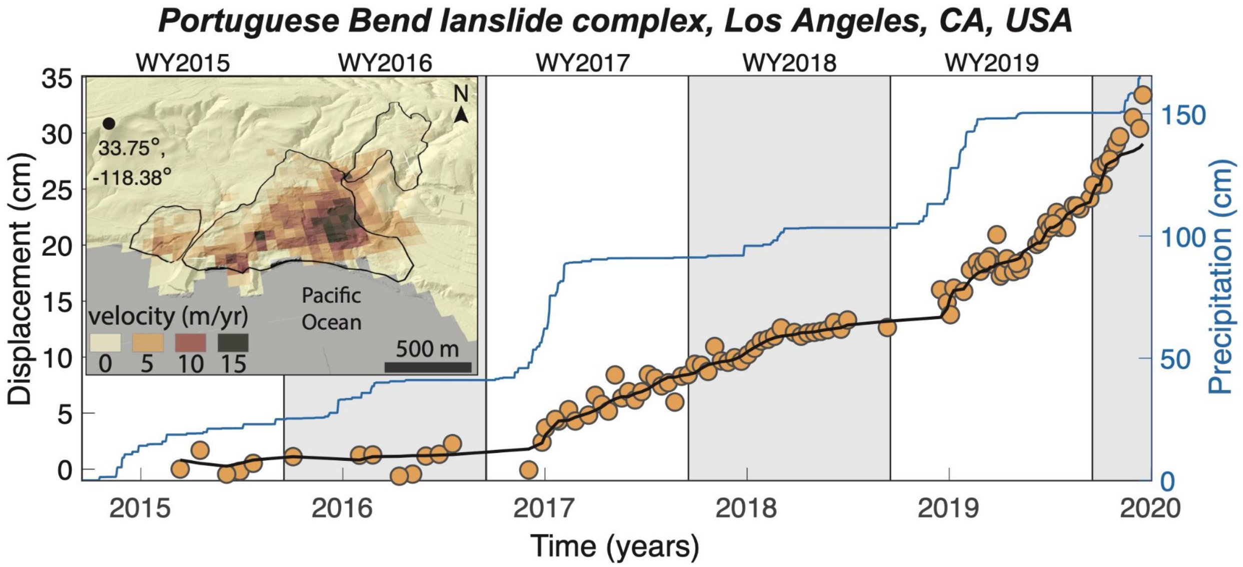

Figure S2. Displacement and precipitation time series for Portuguese Bend landslide. Orange circles show raw InSAR time series and black line shows smoothed time series. Displacement data are projected onto the downslope direction. Blue line shows cumulative precipitation time series. Inset shows oblique view of InSAR velocity map draped over a lidar hillshade. Black circle shows the location of the reference point for the time series and black polygons show active landslide boundaries.

© 2022. California Institute of Technology. Government sponsorship acknowledged.

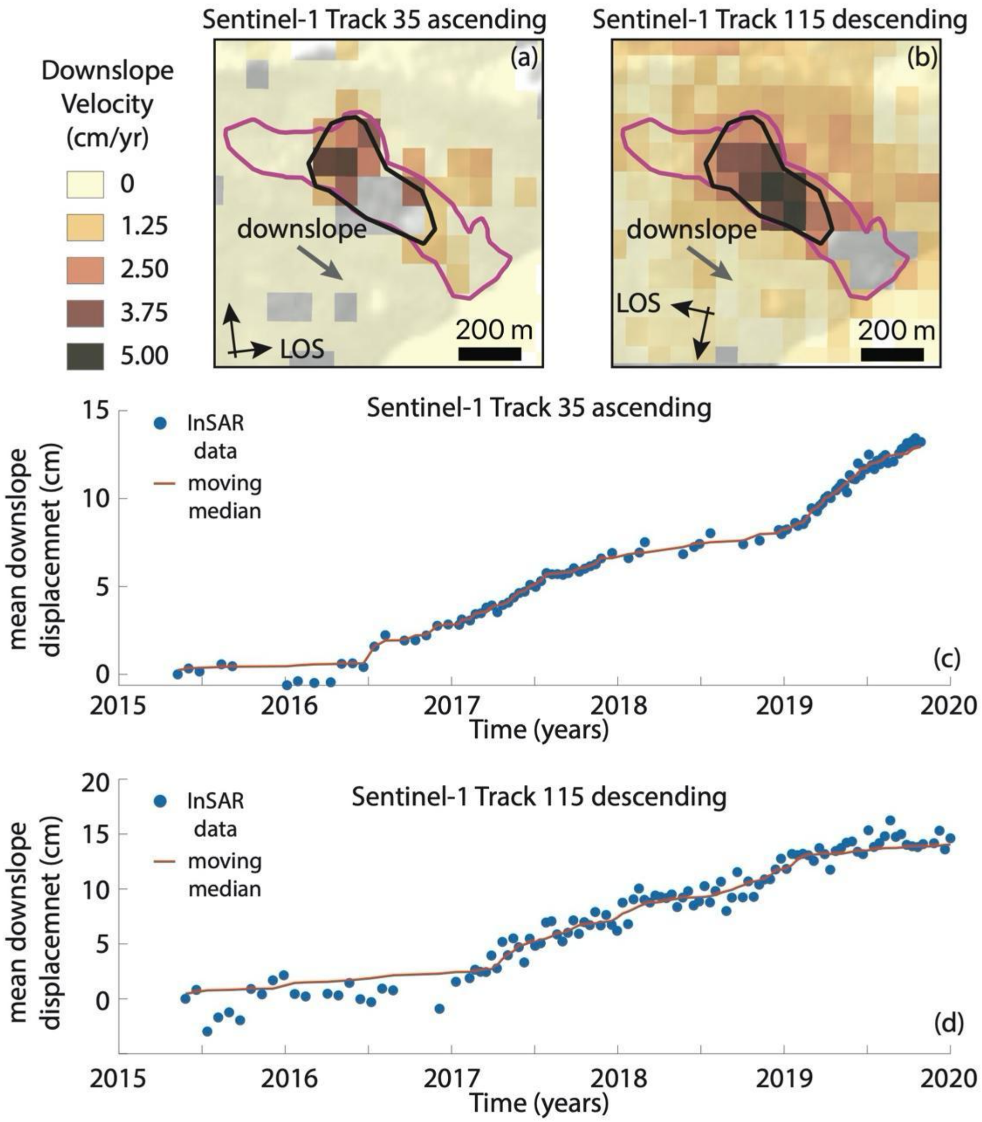

Figure S3. Example ascending and descending deformation maps and time series for a single landslide. (a-b) InSAR velocity maps draped over a hillshade of topography. Red colors show relatively high velocities. Magenta polygon shows the extent of the geomorphic landslide. Black polygon shows the fastest moving zone used to calculate the mean displacement plotted in (c-d). Arrows show downslope direction of landslide, satellite line-of-sight (LOS), and satellite flight heading. (c-d) Mean downslope displacement time series for ascending and descending InSAR data. Blue circles show the raw InSAR data and the orange line shows moving median smoothed time series.

© 2022. California Institute of Technology. Government sponsorship acknowledged.

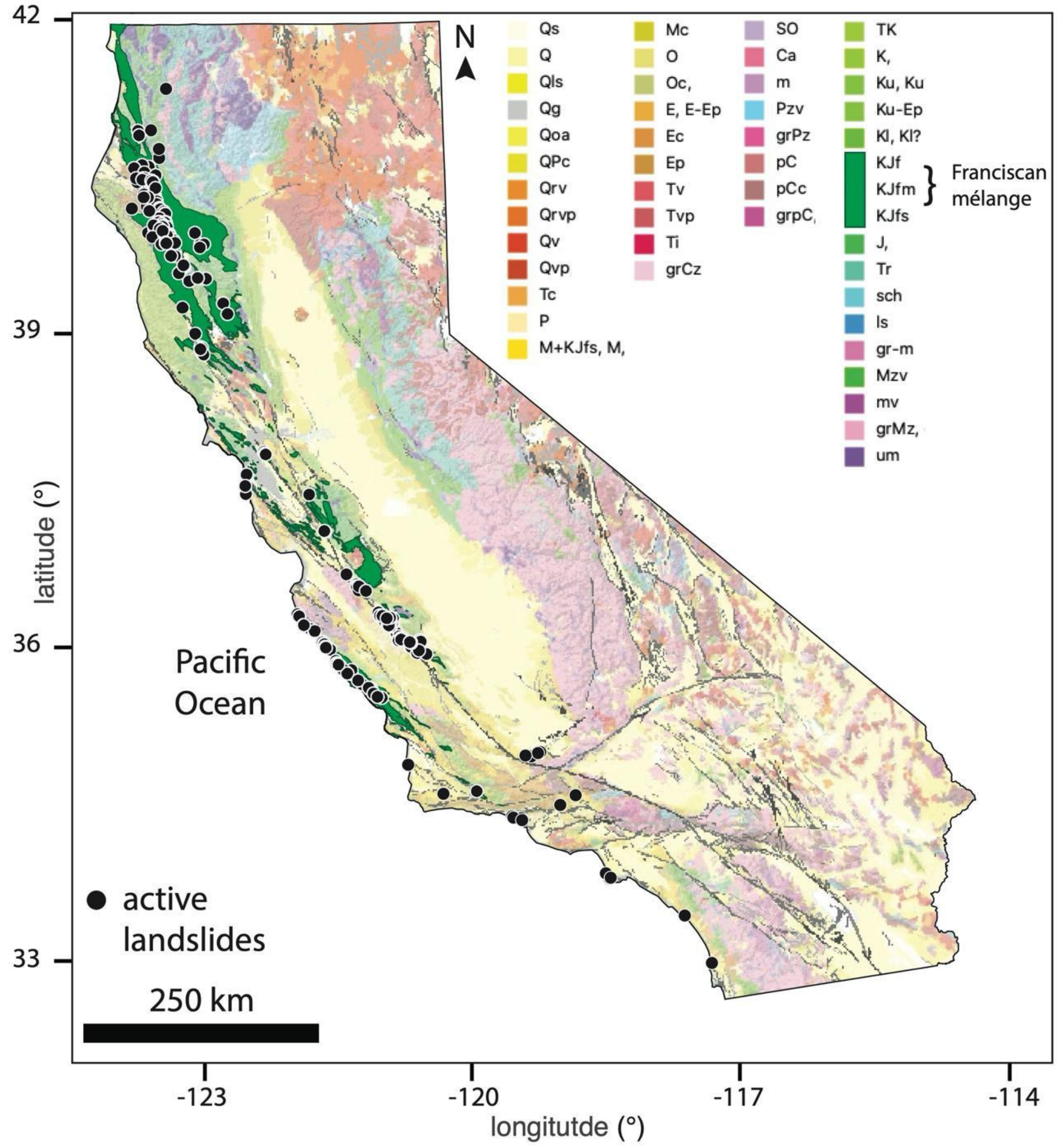

Figure S4. Geologic map of California. Black circles show location of active landslides identified with InSAR data. Data are provided by the California Geologic Survey. For detailed rock type descriptions see list at: https://maps.conservation.ca.gov/cgs/metadata/GDM_002_GMC_750k_v2_metadata.html.

© 2022. California Institute of Technology. Government sponsorship acknowledged.

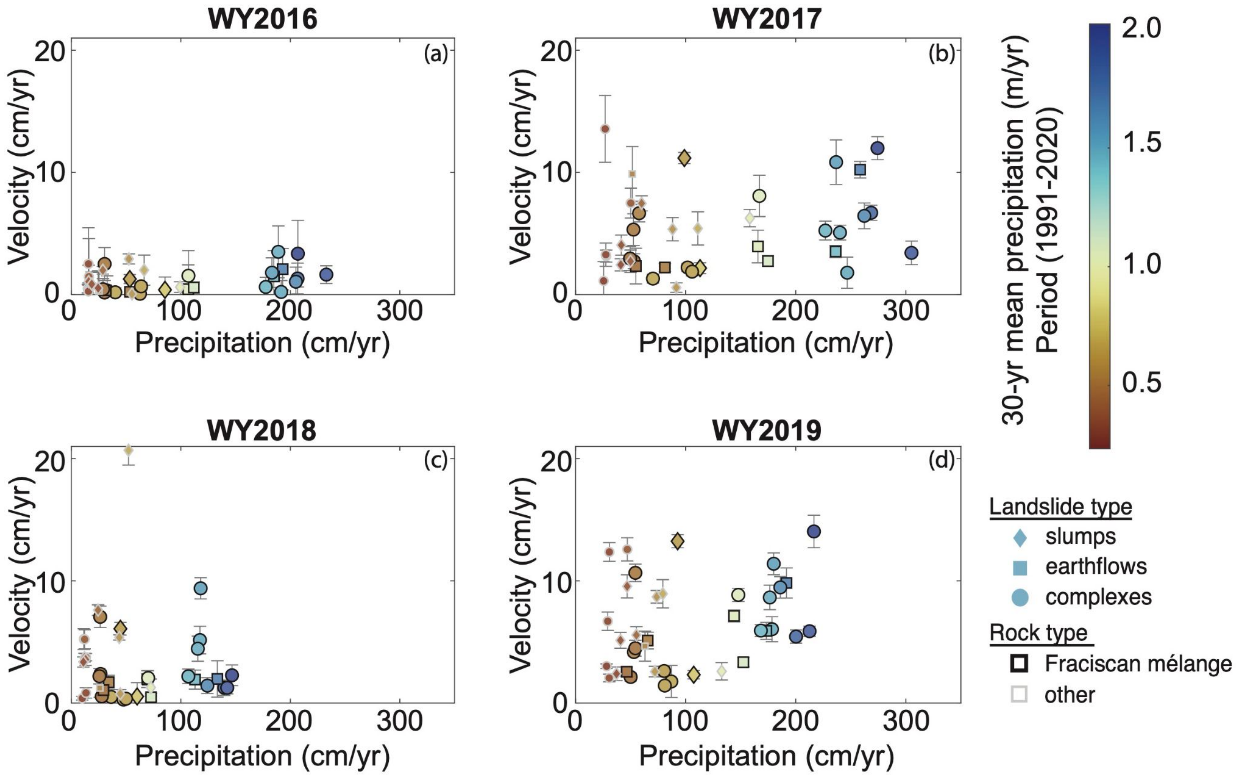

Figure S5. Landslide kinematics in response to changes in precipitation. (a-d) Water year (WY) velocity as a function of WY precipitation for selected landslides. Error bars show the uncertainty in the velocity estimates. Red to blue colors correspond to the 30 year normal precipitation (1991-2020) for each landslide. Symbols correspond to landslide type. Rock type is shown by black or gray symbol border color.

© 2022. California Institute of Technology. Government sponsorship acknowledged.

Supplementary Tables

Captions for Tables S1, S3, S4, S6:

Table S1. List of all InSAR pairs used in study. Columns correspond to the reference acquisition dates, secondary acquisition dates, perpendicular baseline, the timespan between images, and the total number of interferograms used in the time series inversion.

Table S3. Landslide inventory data table. Table includes information for landslide type, host rock type, mean slope angle, landslide area, landslide length, landslide width, centroid location of each landslide, and 30-year mean (WY1990-WY2019) water year precipitation for each landslide.

Table S4. Landslide data table for the 38 selected landslides. Table includes information for landslide name, centroid location, landslide type, host rock type, landslide area, landslide length, landslide width, estimated landslide volume (inventory and by landslide type), estimated landslide thickness (inventory and by landslide type), mean slope angle, downslope aspect direction, InSAR data used for final time series analyses, stable reference point, and velocity and precipitation data. We estimated landslide volume and thickness using published geometric scaling relations for slow-moving landslides shown in Table S2. Volume and thickness estimates are provided for the inventory and for classified landslide type.

Table S6. List of SRTM DEM tiles used in this study.

© 2022. California Institute of Technology. Government sponsorship acknowledged.

category	c_V, best fit intercept	γ, best fit power function exponent	c_h, best fit intercept	ζ, best fit power function exponent
**inventory**	0.2074 (0.0746, 0.5761)	1.306 (1.213, 1.399)	0.2074 (0.0746, 0.5761)	0.3058 (0.2129, 0.3987)
**slumps**	0.0301 (0.0020, 0.4569)	1.493 (1.224, 1.762)	0.0301 (0.0020, 0.4569)	0.4926 (0.2236, 0.7615)
**earthflows**	0.0207 (0.0013, 0.3389)	1.535 (1.273, 1.796)	0.0207 (0.0013, 0.3389)	0.5348 (0.2734, 0.7963)
**complexes**	0.9542 (0.1029, 1.2674)	1.172 (0.9858, 1.357)	0.9542 (0.1029, 1.2674)	0.1716 (-0.0142, 0.3573)

Table S2. Volume-area scaling fit values (with 95% confidence bounds). Table is modified from Handwerger et al., 2021.We show scaling fit values for all slow-moving landslides combined (referred to as "inventory") and by landslide type.

© 2022. California Institute of Technology. Government sponsorship acknowledged.

reference point longitude (degrees)	reference point latitude (degrees)
-116.3914054	34.26675748
-116.5356684	33.8258261
-117.274774	32.844484
-117.646708	34.288767
-117.8200602	33.62590427
-118.3619388	33.76003981
-118.575646	34.042921
-118.67185	34.55515674
-119.632258	37.985456
-120.0213188	39.4384228
-120.021619	39.43817
-120.87114	36.272458
-121.0954394	36.74856042
-121.190797	36.622521
-121.445966	35.876472
-121.536581	36.026733
-121.585882	36.061785
-121.675278	37.344674
-121.7488595	37.46076441
-121.809952	36.26085
-121.8681335	37.15969
-121.886534	36.426898
-122.265977	37.31953
-122.290836	37.926315
-122.483311	37.699138
-123.050031	38.885312
-123.3293693	39.65829982
-123.393747	39.806784
-123.467188	40.106911
-123.800918	40.558561
-123.816244	40.987106

Table S5. Location of stable reference points used for landslide identification during the statewide mapping.

© 2022. California Institute of Technology. Government sponsorship acknowledged.
